# Efficient Anion-Exchange Membranes with Anti-Scaling Properties Obtained by Surface Modification of Commercial Membranes Using a Polyquaternium-22

**DOI:** 10.3390/membranes12111065

**Published:** 2022-10-29

**Authors:** Dmitrii Y. Butylskii, Vasiliy A. Troitskiy, Maria A. Ponomar, Ilya A. Moroz, Konstantin G. Sabbatovskiy, Mikhail V. Sharafan

**Affiliations:** 1 Membrane Institute, Kuban State University, 149 Stavropolskaya St., 350040 Krasnodar, Russia; 2 Frumkin Intstitute of Physical Chemistry and Electrochemistry RAS, 31 Leninsky Prospekt, 119071 Moscow, Russia

**Keywords:** anion-exchange membrane, membrane modification, polyquaternium-22, surface charge, electrodialysis, water splitting, electroconvection, membrane scaling

## Abstract

Anion-exchange membranes modified with a polyquaternium-22 (PQ-22) polymer were studied for their use in electrodialysis. The use of PQ-22 for modification makes it possible to “replace” weakly basic amino groups on the membrane surface with quaternary amino groups. It was found that the content of quaternary amino groups in PQ-22 is higher than the content of carboxyl groups, which is the reason for the effectiveness of this polymer even when modifying Ralex AHM-PES membranes that initially contain only quaternary amino groups. In the case of membranes containing weakly basic amino groups, the PQ-22 polymer modification efficiency is even higher. The surface charge of the modified MA-41P membrane increased, while the limiting current density on the current-voltage curves increased by more than 1.5 times and the plateau length decreased by 2.5 times. These and other characteristics indicate that the rate of water splitting decreased and the electroconvective mixing at the membrane surface intensified, which was confirmed by direct visualization of vortex structures. Increasing the surface charge of the commercial MA-41P anion-exchange membrane, reducing the rate of water splitting, and enhancing electroconvection leads to mitigated scaling on its surface during electrodialysis.

## 1. Introduction

Polymer membranes with functional groups covalently bound to their backbone (i.e., ion-exchange membranes, IEMs) are widely used in numerous engineering applications, such as water desalination, selective extraction of target ions, power generation, sensors, chemical synthesis, etc. [1,2,3,4]. The specific purpose of membranes is determined by the transport properties of both their volume and surface, the control of which is attempted at the stage of membrane synthesis [5,6,7,8]. Since membrane synthesis is a laborious process, changing the physical or chemical properties of the surface of a commercial membrane is often the cheapest and easiest way to achieve the required properties.

For more than 30 years, the main efforts have been focused on the modification of commercial samples of membranes, which has the following main goals: increase in ion-exchange capacity [9,10], control of membrane hydrophilicity concerning both permselectivity and antifouling characteristics [11,12,13,14,15], decrease in diffusion and osmotic permeability [16,17], enhancement of proton or hydroxide conductivity and increase in the thermal stability of membranes [18,19,20], and control of the over-limiting mass-transfer mode (changing the water-splitting rate [15,21,22,23,24] and intensification of the mass transfer [25,26,27,28]). Therefore, different surface modification methods are reported in the literature, such as layer-by-layer modification [15,29,30], dip coating [31,32], polymer blending [33], covalent cross-linking [34,35], electrodeposition [36,37], electrospinning [38,39] and structural membrane fabricating [40], and organic-inorganic composite material production [41,42].

To determine the most appropriate modification procedure, in addition to the knowledge of the properties of available modifying agents, a comprehensive understanding of the physicochemical properties of membranes is required. The study of the structure–property relationships of ion-exchange membranes is one of the most important directions forming the fundamental basis for developing new, more perfect membranes [43,44,45].

It is known that any membrane surface heterogeneity (electrical or geometric) causes the appearance of a tangential component of the electric force, which stimulates the development of electroconvection (EC) [46,47,48]. It can significantly intensify the mass transport, leading to a growth in the limiting and over-limiting current densities [25,49,50], a change in the shape of chronopotentiograms [51,52,53,54], and the presence of electrochemical impedance spectra [55]. Another key factor in the development of electroconvection (electro-osmosis of the first kind, according to terminology used in [47,56]) is the value of the membrane surface charge [57,58,59]. According to I. Rubinstein and B. Zaltzman [57,58], with an increase in the surface charge, electroconvection should increase.

However, there are many difficulties in measuring the surface charge (or rather, streaming potential) of polymer membranes and there are not many works devoted to this in the literature [60,61,62]. The situation is complicated by the fact that the surface charge of the membrane used in electrodialysis varies depending on the given current density. This is due to the occurrence of water splitting at the interface, where the functional groups (responsible for the surface charge) are directly involved in the protonation and deprotonation reactions. As a result, a current-induced discharge may occur [63] when the effective surface charge is lower than the nominal one.

It was recently shown by Nikonenko et al. [59,64,65] that the electrochemical characteristics of heterogeneous anion-exchange membranes can reach those of homogeneous membranes due to the properties of the surface (electrical and geometric heterogeneities) and the change in its charge. They described [64,65] modified membranes, which were obtained by treating the surface of the commercial membrane with a solution of a bifunctional polymer (DADMAC and acrylic or maleic acid copolymer).

In general, a bifunctional polymer is a copolymer of an organic base and an organic acid containing both functional groups of one and the other reagent. The use of such polymers to modify anion-exchange membranes makes it possible to replace functional tertiary and secondary amino groups with quaternary ones while maintaining membrane surface properties [64]. Similar bifunctional polymers were used as membrane modifiers in other studies [66,67,68]. In all cases, an improvement in the electrochemical properties of the membranes was also observed. However, the layer-by-layer method of modification is most often used in membrane science, including the application of polymers containing quaternary amino groups. This makes it possible to improve the antifouling performance [69] and to increase the permselectivity of membranes for specific ions [36,70,71,72].

This work also investigates the properties of membranes modified with a bifunctional polymer containing quaternary amino groups. However, in this case, the polyquaternium-22 (PQ-22) commercial polymer, which is defined as a copolymer of acrylic acid and diallyldimethylammonium chloride, is used as the modifier. Efforts were focused on not only investigating improved mass-transfer properties and reduced water-splitting rates on the modified membranes but also on studying the reasons for the effectiveness of the PQ-22 polymer as the modifier of anion-exchange membranes. In addition, commercial and modified membranes were tested for scaling resistance.

## 2. Materials and Methods

### 2.1. Membranes

Heterogeneous anion-exchange MA-41P and Ralex AHM-PES membranes—which differ in surface structure and chemical composition—and their modified samples (MA-41Pmod and Ralex AHM-PESmod) were studied.

The Ralex AMH-PES heterogeneous membrane (Mega a.s., Straz pod Ralskem, Czech Republic) consists of finely ground Lewatit M500 anion-exchange resin (*d_av_* = 5 μm) and polyethylene, the proportion of which is 2 times less compared to the resin [73]. To impart mechanical strength to the resulting composite membrane, the Ulester 32S reinforcing mesh is pressed into it from both sides after heating the mixture of resin and the inert binder [74].

The MA-41P heterogeneous membrane (JCC Shchekinoazot, Pervomayskiy, Russia) contains 60% particles of AV-17 anion-exchange resin (*d_av_* = 18 μm) and polyethylene. The membrane is reinforced with a cloth of Nylon 6 fibers. In contrast to the Ralex AMH-PES membrane, the MA-41P membrane, in addition to quaternary amino groups, contains a small amount of secondary and tertiary amines as functional groups [75]. Due to the high content of the inert binder and the large average particle diameter of the ion exchanger in the MA-41P composition compared to the Ralex AMH-PES membrane, the surface and volume of this membrane are extremely heterogeneous. The fraction of the conductive surface is only 0.35 [65]. The data on the membranes under study are summarized in Table 1.

The MA-41Pmod and Ralex AHM-PESmod membranes are made by modifying the membrane surface with polyquaternium-22 (CAS No. 53694-17-0, Career Henan Chemical, Zhengzhou, China). The MA-41Pmod and Ralex AHM-PESmod membranes were obtained by soaking the pristine membranes for 8 h in a 2.5% PQ-22 aqueous solution. PQ-22 is defined as a copolymer of acrylic acid and diallyldimethylammonium chloride (DADMAC). Modification of the membranes does not lead to a change in the parameters described in Table 1 for the pristine membranes that exceed the confidence intervals. This was also noted in [64].

**Figure 1:**
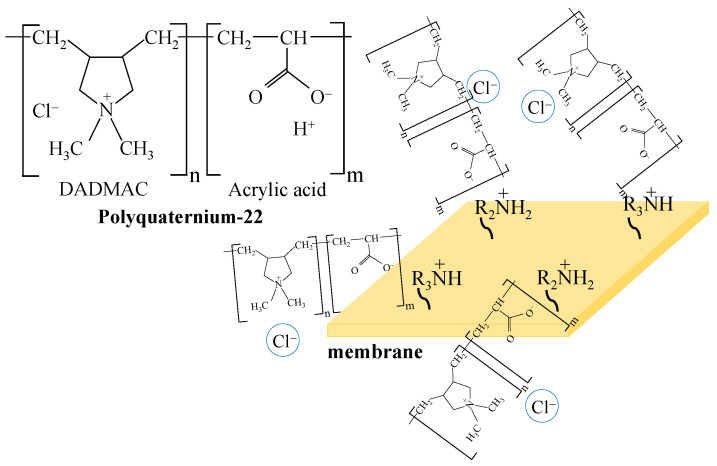
Interaction of modifier with functional groups on the surface of MA-41P membrane.

The corresponding structural formula and the scheme of interaction of PQ-22 with the MA-41P membrane surface, for example, are shown in Figure 1. Polymeric quaternary ammonium salts (polyquaterniums), such as PQ-22, are increasingly used in industries, particularly as conditioners in cosmetics. The PQ-22 polymer contains quaternary amino groups, in which nitrogen is biodentically bound to the alkyl matrix. The carboxyl groups of the modifier ensure its interaction with fixed, positively charged groups on the membrane surface.

### 2.2. Methods

The qualitative and quantitative composition of the modifier was determined by potentiometric titration and Mohr titration, as well as FTIR spectroscopy using a Vertex 70 FT-IR Spectrometer (Bruker Corporation, Bremen, Germany).

The electrochemical characteristics of the studied membranes were measured in 0.02 M sodium chloride solution. This binary electrolyte is most often used for testing ion-exchange membranes if there is no connection to a specific process. This concentration was selected since, at this concentration, the co-ion transfer through the membrane is negligible for most membranes; however, current-induced phenomena (water dissociation and electroconvection, which occur in real electrodialysis) are well developed.

### 2.3. Flow-Through Electrodialysis Cell for Measuring Electrochemical Characteristics

**Figure 2:**
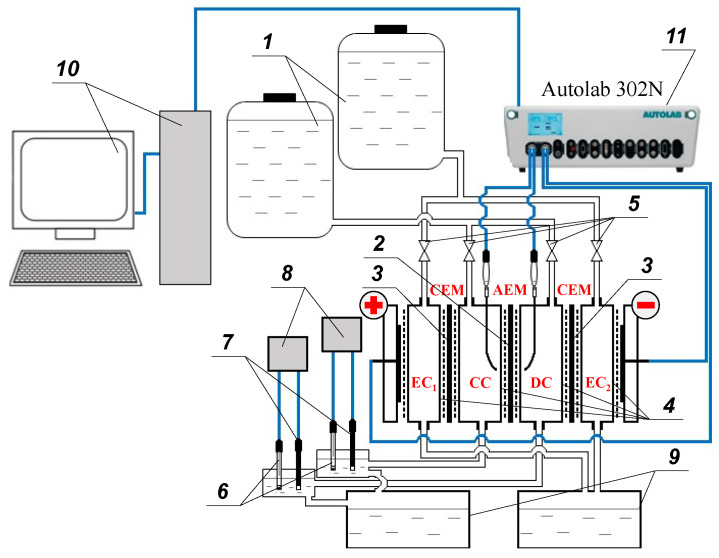
Schematic diagram of a setup for studying the electrochemical characteristics of anion-exchange membranes.

A flow-through four-chamber electrodialysis cell with setup (Figure 2) and an Autolab PGSTAT 302N potentiostat-galvanostat with a FRA32M impedance measurement module were used to record current-voltage characteristics, CVC, and impedance spectra. For ease of comparison of the results, the experiments were carried out at current densities normalized to the values of the theoretical current density, *i*_lim_, obtained by the Lévêque equation within the framework of the convective-diffusion model (please see Supplementary Materials). The calculated value of *i*_lim_ is equal 3.0 mA/cm^2^.

An electrolyte solution (0.02 M NaCl, pH 5.6 ± 0.1) from two containers (1) entered a four-chamber electrodialysis flow cell. The investigated anion-exchange membrane (AEM) (2) (working area is 4 cm^2^) and auxiliary cation-exchange membranes (CEM) (3) (MK-40, Shchekinoazot), using frames (4), formed a concentration chamber (CC), a desalination chamber (DC), and electrode chambers (EC_1_ and EC_2_) of the electrodialysis cell, in which the flow rate of the solution was set using taps (5) and amounted to 27 mL/min (≈0.4 cm/s). At the outlet of the CC and DC, the composition of the solution was continuously monitored using combined pH electrodes (6) and conductivity sensors (7) connected to Expert-001 pH meters and Expert-002 conductometers (Expert-Econix, Russia) (8), respectively. Then, the solutions entered two tanks (9), in which the solutions from DC and CC and from EC_1_ and EC_2_, respectively, were mixed. The spent solution from the tanks (9) was supplied using pumps in the containers (1), realizing a circulation-type hydraulic system. The solution (V = 15 L) was changed approximately every 10 h of operation.

The electrochemical characteristics of the studied AEMs were obtained using a computer-controlled (10) Autolab PGSTAT 302N potentiostat-galvanostat (11) with an FRA32M module for measuring impedance. The device polarized the electrodialysis cell through platinum electrodes and recorded the response of the membrane under study using silver chloride measuring electrodes connected to Luggin capillaries supplying both sides of the studied AEM at a distance of about 0.8 mm from its surface.

### 2.4. Streaming and Zeta Potential of Membrane Surface

To determine the surface charge of the membranes, we measured the streaming potential, (Δ*φ*)*_sp_*, in a gap cell when the solution was pressed along the slit, the walls of which were formed by the surfaces of two identical samples of the membrane under study [76]. The streaming potential measurements for all samples were also carried out in 0.02 M sodium chloride solution at pH = 6.2 ± 0.1.

**Figure 3:**
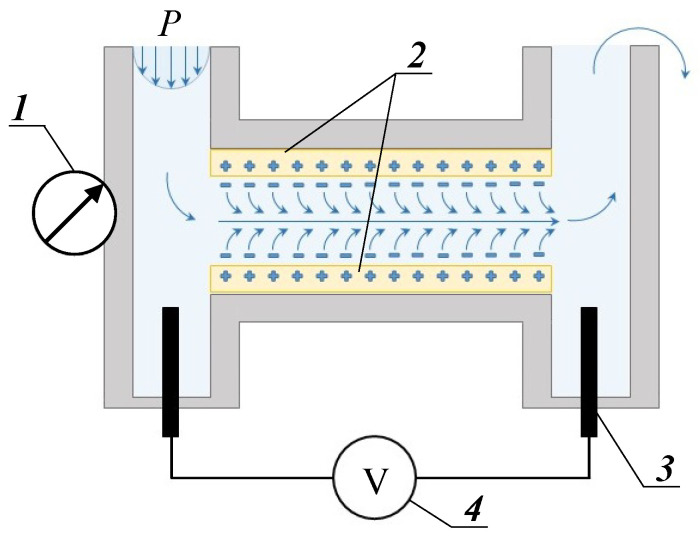
Scheme of the gap cell for measurement of streaming potential: 1—pressure gauge, 2—studied samples, 3—Ag/AgCl electrode, 4—multimeter. Reprinted with permission from Ref. [59]. Copyright © 2017, Elsevier.

The gap cell used for measuring the streaming potential of the studied anion-exchange membranes is described in [76] and schematically shown in Figure 3. It is similar to that applied in the Anton Paar SurPass 3 electrokinetic analyzer. The latter one was employed by Yaroshchuk and Luxbacher [77] for measuring external and internal (inside membrane pores) zeta potential, as well as by Sedkaoui et al. [78], who developed a promising method for determining the lateral conductivity of ion-exchange membranes from the measurements of the streaming current and streaming potential. In our cell, the two samples under study form a rectangular slit channel of 25 ± 0.2 mm length, 1.8 ± 0.2 mm width, and 100 ± 1 μm height. The experiments were conducted at 25 °C, using a 0.02 M NaCl solution pumped with a linear velocity of 530 ± 30 cm·s^−1^.

Streaming potential, ∆(*φ*)*_sp_*, was registered in the range of pressure drops between 0.05 and 1.0 bar with the help of two Ag/AgCl electrodes (Figure 3) using a GW Instek multimeter connected through a U5-12 amplifier.

To express the zeta potential, ζ, from the measured tangential streaming potential, ∆(*φ*)*_sp_*, of membranes, the classical Helmholtz–Smoluchowski equation [79,80,81,82] was applied:

(1) $$\zeta =\frac{{\left(\Delta \varphi \right)}_{sp}}{\Delta P}\frac{\eta \kappa_{Bulk}}{\varepsilon \varepsilon_{0}}$$

where $\kappa_{Bulk}$ is the conductivity of the solution feeding the gap cell, ∆(*φ*)*_sp_* is the tangential streaming potential, ∆*P* is the pressure drop over the channel formed by the membranes, η is dynamic viscosity of the solution, ε is the relative permittivity of the liquid, and $\varepsilon_{0}$ is the electrical permittivity of the vacuum.

The surface charge, *σ*, of the membrane under study is estimated using the Grahame equation [83]:

(2) $$\sigma =\sqrt{8\varepsilon \varepsilon_{0}CRT}\sinh\left(\frac{\zeta F}{2RT}\right)$$

### 2.5. Visualization of Electroconvective Vortices

**Figure 4:**
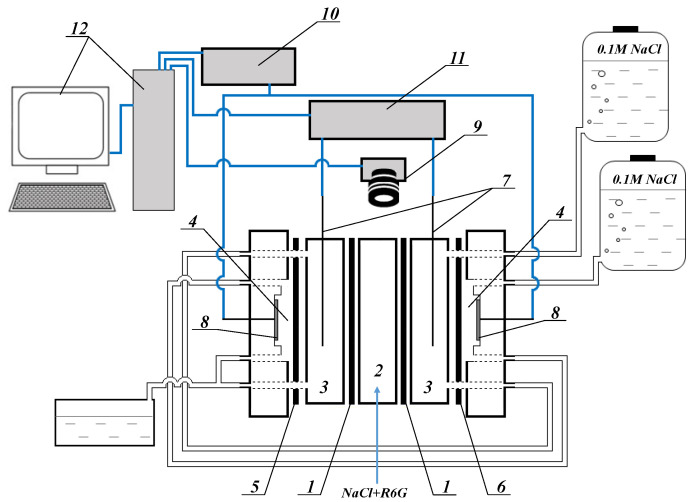
The scheme of an experimental setup for the visualization of vortex structures near anion-exchange membranes.

To visualize vortex structures in a lean solution near the membrane surface, the setup (Figure 4) and method are described below. Two pieces of the studied anion-exchange membrane (1) form a chamber (2) in which the depleted boundary is studied. Buffer (3) and electrode (4) chambers are separated by auxiliary membranes MK-40 (5) and MA-41 (6), respectively. The length of these compartments is 0.53 cm and the intermembrane distance is 0.32 cm. Luggin capillaries (7) are inserted into buffer (3) chambers from both sides of the studied AEMs (1). The capillaries, in turn, are connected to the measuring Ag/AgCl electrodes. The polarizing electrodes (8) are located in the electrode chambers (4). A 0.02 M NaCl + 10 µM Rhodamine 6G (R6G) solution is pumped through the chamber (2). A 0.1 M sodium chloride solution is circulated through the buffer (3) and electrode (4) chambers. The linear velocity of the pumped solutions is 0.07 cm·s^−1^ in the chamber (2), 0.14 cm·s^−1^ in the buffer chambers (3), and 19 cm·s^−1^ in the electrode chambers.

At pH close to 6, R6G dissociates into anion Cl^−^ and cation R6G^+^ [84], with the latter fluorescing in the wavelength range of 540–630 nm. Video recording of the fluorescence of R6G^+^ in the depleted solution near the membrane surface was carried out by a CMOS camera with a magnifying lens 180×, equipped with a fluorescent device (9). The current density in the membrane system was set by a Keithley sourcemeter 2400 (10). The potential drop between the Luggin capillaries (7) is measured by a Keithley multimeter 2010 voltmeter (11). All devices are controlled by a personal computer (12).

Since the area of the depleted solution near the anion-exchange membranes was under study, the concentration of the fluorescent cation (as well as Na^+^) is higher here than in the channel. The low fluorescent R6G^+^ content gives the solution a black color when the interface is visualized [85]. The resolution of the digital optical system makes it possible to register the appearance of vortex structures with a diameter of about 20 microns or more. Digital video recording was carried out in over-limiting current modes.

The main feature of the device is a planar electrodialyzer, of which the length of the chambers and the intermembrane distance are much less than the analogous parameters of an electrodialyzer for studying electrochemical properties. This makes it possible to monitor the development of vortex flows on the membrane surface in a narrow section without their integration.

## 3. Results and Discussion

### 3.1. Electrochemical Characteristics of Pristine and Modified Membranes

**Figure 5:**
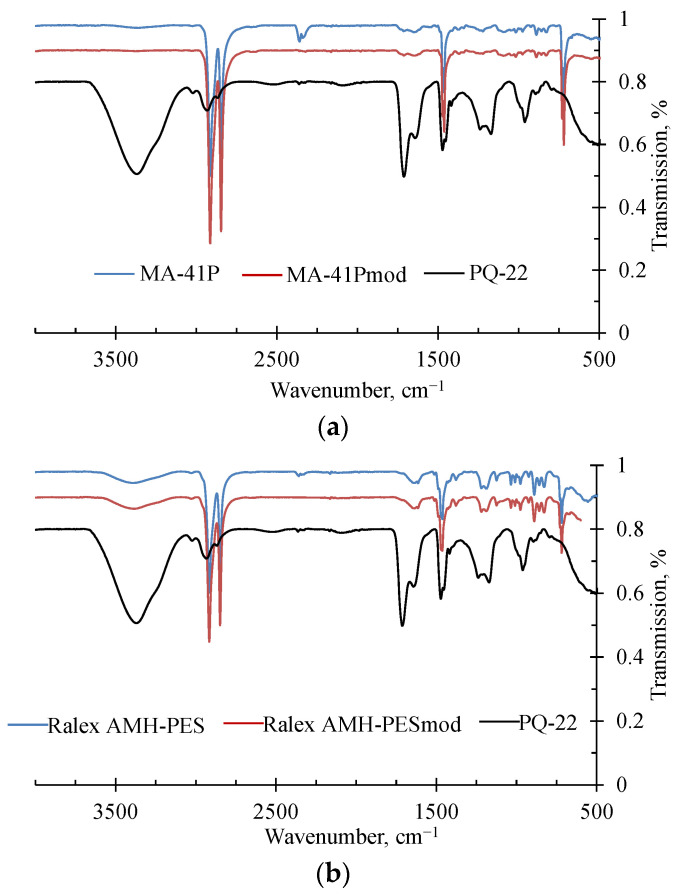
The FT-IR spectra of MA-41P and MA-41Pmod (**a**), Ralex AHM-PES and Ralex AHM-PESmod samples (**b**), and dry PQ-22 polymer film.

The study of commercial MA-41P and Ralex AHM-PES and modified MA-41Pmod and Ralex AHM-PESmod samples by FT-IR spectroscopy showed that the corresponding samples were almost indistinguishable (Figure 5). Unfortunately, the quaternary amino groups do not have a characteristic peak. In addition to the characteristic C–H peaks (2900 and 1465 cm^−1^) of the polyethylene binder, the spectra of the membranes contain peaks corresponding to the N-H bond (1200 and 730 cm^−1^). The application of the modifier to the surface of the membranes does not lead to the appearance of additional peaks but only slightly increases the intensity of the present peaks. This also explains the fact that membrane modification does not change the parameters (water content, exchange capacity, and conductivity) described in Table 1.

**Figure 6:**
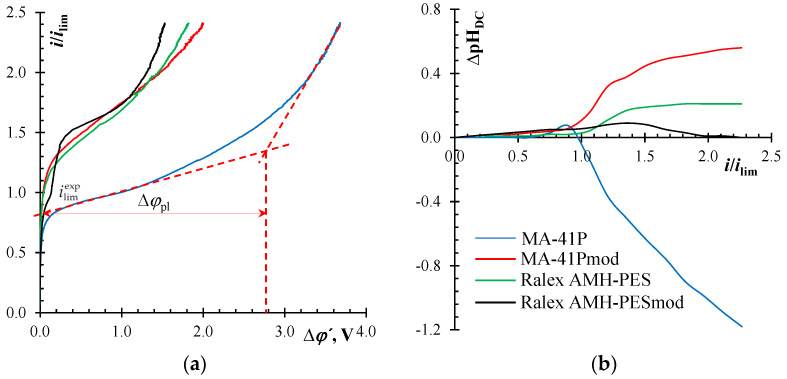
Current-voltage curves of the anion-exchange membranes under study (**a**), as well as differences in the pH of the inlet and outlet solution in the desalination chamber (∆pH_DC_) (**b**). ∆*φ*′ is reduced potential drop (see Appendix A).

As it was established in this work, the electrochemical characteristics of the heterogeneous MA-41P membrane are much worse than those for the Ralex AHM-PES membrane. The value of the limiting current density found experimentally from current-voltage curves, CVCs, $i_{\lim}^{\exp}$, for the pristine MA-41P membrane is less than the calculated value (3.0 mA/cm^2^) and almost 1.5 times less than for the pristine Ralex AHM-PES membrane (Figure 6a).

The reason for the increase in the experimental limiting current over the calculated value for Ralex AHM-PES could be the equilibrium electroconvection (EC), also called electro-osmosis of the first kind [56,57]. This has been previously discussed in the case of such modified membranes [32,65]. This is confirmed by the results of measuring the zeta potential and, as a consequence, by the large surface charge of the Ralex AHM-PES membrane in comparison with MA-41P (Table 2). Its rather high values are a very important condition for the development of the equilibrium EC.

Undoubtedly, equilibrium electroconvection occurs under the action of a tangential electric force on the space charge of an equilibrium electrical double layer, provided that the membrane has an ideal surface and is selectively permeable [57,86]. In the case of real membranes with roughness, waviness, and inhomogeneity, etc., it is difficult to isolate this phenomenon [27,50,87,88]. This is due to the fact that non-equilibrium EC, also called electro-osmosis of the second kind, and that which develops in over-limiting current modes [27,47,56], also occurred well in the case of the Ralex AHM-PES membrane. An indicator of its development is the “plateau length”, ∆*φ*_pl_, of a CVC. It is considered to be a threshold potential drop at which non-equilibrium EC occurs. The “plateau length” is 2.5 times less for the pristine Ralex AHM-PES membrane compared to MA-41P (Figure 6a).

The rate of development of non-equilibrium EC can also be deduced from the change in the pH value of the solution entering the desalination chamber (Figure 6b). pH values were recorded during the measurement of CVCs (Figure 6a). It is known from the literature that a high rate of water splitting can be caused by a low intensity of non-equilibrium EC and vice versa [89,90,91]. At currents above the limiting value (>1.0 *i*/*i*_lim_), the presence of a certain amount of tertiary and secondary amines in the composition of MA-41P leads to intensive generation of H^+^ and OH^−^ ions (water splitting), according to [92,93,94]. This can be concluded from the results of measuring the difference in pH (∆pH) between the solution coming out and the solution entering the desalination chamber (ΔpH = pH_out_ − pH_in_). There is significant acidification of the diluate (Figure 6b). This means that the water-splitting rate on the anion-exchange membrane is higher than on the cation-exchange membrane forming the desalination chamber [95]. In the case of the Ralex AHM-PES membrane, the opposite situation develops (water-splitting rate on the anion-exchange membrane is lower than on the cation-exchange membrane), which is confirmed by the stable alkalinization of the solution entering the desalination chamber at *i* > *i*_lim_.

Although the ∆pH value depends on the rate of water splitting at both membranes forming the desalination chamber, this is an easy method of comparison if the second membrane forming the corresponding chamber is the same. Its rate can be estimated only on the membrane under study by the method of electrochemical impedance spectroscopy. The parameters of the so-called the Gerischer impedance arc, which appears in the mid-frequency region (10–10^3^ Hz) of the frequency set, correspond to this phenomenon. In the general case, the reason for the appearance of this arc is an extra amount of charge carriers ejected into the solution as a result of a chemical reaction. In the case of the considered membrane system, only the water-splitting reaction at the membrane-solution boundary is the reason for the appearance of an extra amount of charge carriers [96,97,98].

**Figure 7:**
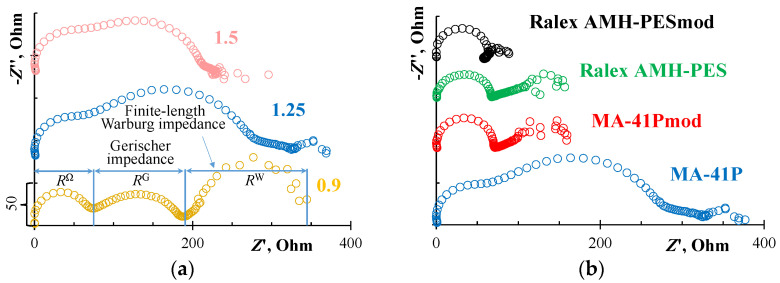
Electrochemical impedance spectra of MA-41P (**a**) at different values of biased direct current (0.9, 1.25, 1.5 *i*_lim_) and spectra of MA-41P, MA-41Pmod, Ralex AMH-PES, and Ralex AMH-PESmod at 1.25 *i*_lim_ (**b**).

Figure 7a shows that in the case of the MA-41P membrane at 0.9 *i*_lim_, the impedance spectrum on the complex Argand plane has a recognizable shape for an ion-exchange membrane. There are high-frequency (1–500 kHz) arcs, the active resistance of which is indicated by *R*^Ώ^, a Gerischer arc (*R*^G^), and a low-frequency (0.003–10 Hz) Warburg arc (*R*^W^).

Analyzing the shape of the spectra depending on the value of the DC bias, it can be seen that, with its increase, the effective resistance of chemical reaction increases, judging by *R^G^*. At 1.5 *i*_lim_, the Gerischer arc partially swallows up the other arcs. These results are in positive agreement with our earlier results [32].

Thus, the water splitting at the pristine MA-41P membrane begins at currents below *i*_lim_ and rapidly accelerates with an increase in the DC bias due to the surface properties (a large proportion of non-conductive regions contributes to the manifestation of the “funnel effect” [51]) and the chemical composition of its functional groups (a small amount of secondary and tertiary amines are present). On the contrary, the Ralex AMH-PES membrane is characterized by the absence of the Gerischer arc at 1.25 *i*_lim_ (Figure 7b) and therefore the water-splitting reaction, which is consistent with the results obtained by other methods (Figure 6, Table 2).

Modification of the surface of both the MA-41P membrane and the Ralex AHM-PES membrane leads to an increase in the limiting current density (Figure 6a). The rate of water splitting at the MA-41Pmod membrane is significantly lower than on the pristine membrane (Figure 6b). This is also confirmed by the results obtained by the method of EIS (Figure 7b). After modification, only the high-frequency and Warburg arcs remain in the spectra, which indicate the absence of a chemical reaction. There are no big differences between the pristine and modified Ralex AHM-PES membranes, because the original membrane initially contained only quaternary amino groups.

It should be noted that the properties of MA-41Pmod are similar to those of the pristine Ralex AHM-PES membrane due to an increase in the surface charge (Table 2) and, as described above, the intensification of equilibrium electroconvection. An increase in the surface charge of the MA-41Pmod membrane as compared to the pristine MA-41P indicates the nonequivalent substitution of secondary and tertiary amino groups on the surface of modified membrane. Indeed, the study of the chemical composition of the modifier by potentiometric titration and Mohr titration methods made it possible to establish that the concentration of carboxyl groups in its structure is 2.45 ± 0.04 mmol/g_PQ-22(dry)_, and the concentration of chlorides, which are counterions of quaternary amino groups, is 5.0 ± 0.2 mmol/g_PQ-22(dry)_. This means that, in reality, the structure of the polymer chain differs from that shown in Figure 1. There are several fragments containing a quaternary amino group (DADMAC) for each fragment containing one carboxyl group (acrylic acid). This phenomenon is difficult to evaluate by other techniques due to difficulties in determining the content of quaternary amino groups on the membrane surface. However, this is also confirmed by the change in the electrochemical properties of the Ralex AHM-PES membrane after modification, as well as by an increase in its surface charge (Figure 6a, Table 2). If the ratio of negatively charged carboxyl groups and positively charged amino groups in the modifier were different, the surface charge of the modified membranes would change accordingly: it would remain the same with an equivalent amount of DADMAC and acrylic acid and decrease with an excess of acrylic acid fragments.

It should be noted that other bifunctional polymers similar to PQ-22 also exhibit inequivalence when modifying anion-exchange membranes, which was established by indirect measurements in the following papers [32,64]. Measuring the streaming potential before and after modification is perhaps the only direct method that can be used to prove this. On the other hand, this phenomenon is indirectly confirmed by the EIS results.

The use of PQ-22 insignificantly reduces the Ohmic resistance, *R*^Ώ^, of the studied membranes and noticeably increases the effective electrical capacitance of their interface (Table 3). Note that the *R*^Ώ^ parameter includes the Ohmic resistance of the membrane under study, the resistance of the boundary diffusion layers (BDL), and the solution between the measuring electrodes, including the resistance of the electrodes themselves. The capacity of the electric double layers is dominant in the given frequency range (the calculation method is described in Appendix A). It is known from studies in the field of electrode kinetics that the capacitance of the interphase boundary increases with an increasing electric charge of the dense part of the electric double layer [96,99]. This means that the modification of membranes with PQ-22 leads to an increase in the electric charge of the surface as compared to the pristine membranes.

To conclude the above, we can say that the modification of the commercial membranes leads to a noticeable increase in $i_{\lim}^{\exp}$ and a shift in the pH of the diluate solution to a more alkaline region compared to the pristine membranes. Thus, intensity of the EC, which occurs as electro-osmosis of the first kind, increases upon modification, which is associated with an increase in the surface charge. However, the intensity of EC, which appears as electro-osmosis of the second kind, also increases, which is associated with the replacement of secondary and tertiary amino groups on the membrane surface with quaternary ones and a subsequent decrease in the rate of water splitting.

### 3.2. Analysis of the Visualization of the Formation of Electroconvective Vortices

**Figure 8:**
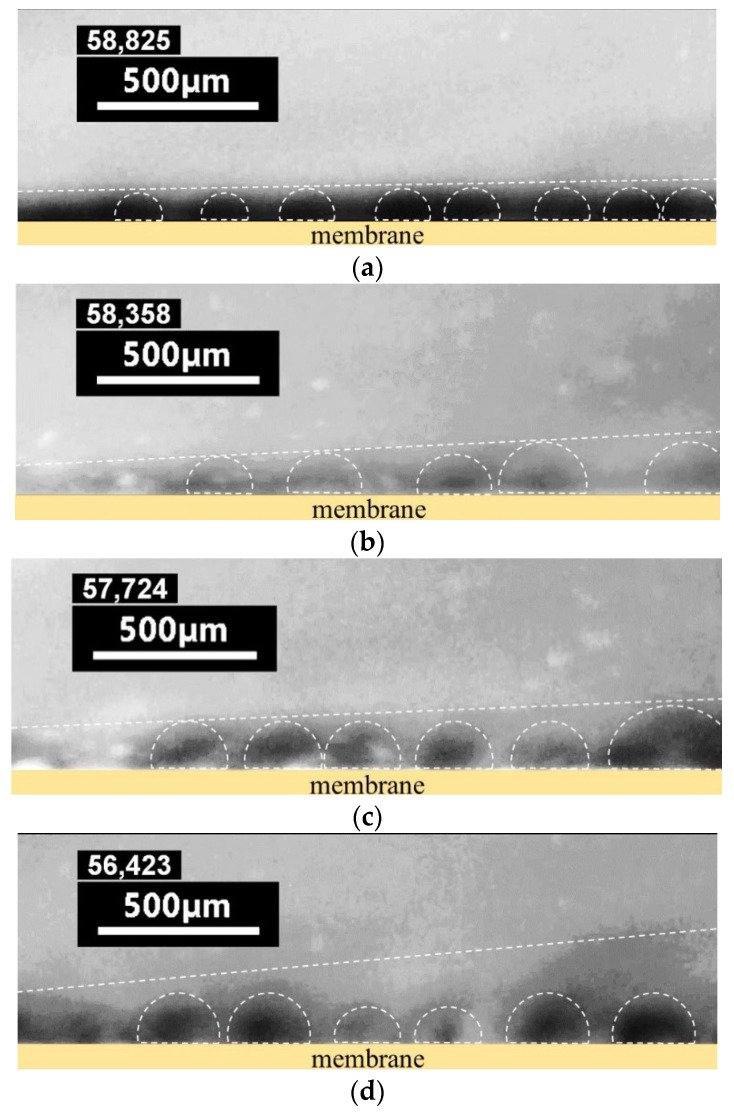
Visualization EC vortexes near the MA-41P (**a**), MA-41Pmod (**b**), Ralex AMH-PES (**c**), and Ralex AMH-PESmod (**d**) membranes during electrodialysis. Supplementary Videos S1–S4 match the same membranes in the same order. Dark areas correspond to a low concentration of rhodamine. The vortexes are circled for clarity.

We visualized the vortex structures that appear in a depleted solution near the anion-exchange membranes under study in an over-limiting current mode. The snapshots are shown in Figure 8. Please refer to Supplementary Videos to track the process of vortex formation over time. The presented results were obtained at the same current density only 2–3 times higher than the limiting current density, which was estimated from the previously obtained experimental CVCs.

In the case of the pristine MA-41P membrane (Figure 8a, Video S1), after switching on the current, the counterion concentration (Cl^−^) near its surface rapidly decreases, which can be estimated from the rhodamine concentration. After 50 s, the concentration profile stabilizes but the vortices are either absent or stand still. This is the expected result. As mentioned above, under conditions of a high rate of water splitting at this membrane, the intensity of non-equilibrium EC is greatly reduced [89,91]. After the modification of this membrane (Figure 8b, Video S2), when the water-splitting rate decreases, vortices form faster (about 20s) after switching on the current and are comparable in intensity to the Ralex AMH-PES membrane (Figure 8c, Video S3). Thus, the modification leads to an increase in the rate of delivery of a more concentrated solution to the membrane surface. As for the Ralex AMH-PESmod (Figure 8d, Video S4) membrane, there are no significant differences in vortex size compared to the pristine Ralex AMH-PES membrane, which is probably due to the initially high content of quaternary amino groups in its composition and optimal surface morphology. In this case, the thickness of the EC mixing zone noticeably increases.

### 3.3. Evaluation of Scale Resistance of Anion-Exchange Membranes

We have tested commercial and modified membranes for scale resistance to demonstrate the effectiveness of membranes after modification in application aspects. For testing, an artificial solution was used, corresponding to natural waters in terms of component composition (Li^+^~0.25 g/L, Na^+^~1.15 g/L, K^+^~2.5 g/L, Ca^2+^~1.5 g/L, Mg^2+^~0.3 g/L, HCO_3_^−^~4.3 × 10^−3^ g/L, Cl^−^~8.86 g/L, and pH 3.9). The qualitative chemical composition of the artificial solution corresponds to the composition of mine water from an oil and gas production region in Eastern Siberia (Russia). The economic importance of the waters of this region is due to the high content of lithium (up to 0.5 g/L) [95,100]. However, high concentrations of calcium and magnesium lead to high energy and resource costs during its extraction. Reducing their concentration by hydrometallurgical or membrane methods also leads to a decrease in the concentration of singly charged ions, including lithium. Therefore, the quantitative composition of the artificial solution differs from the quantitative composition of the natural mine water solution selected as a comparison [95].

**Figure 9:**
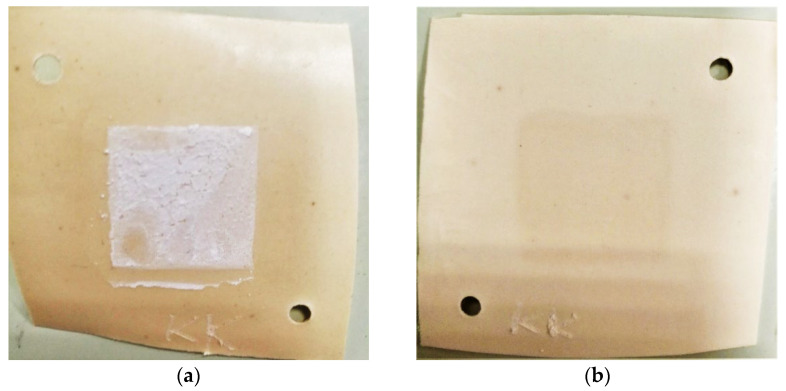

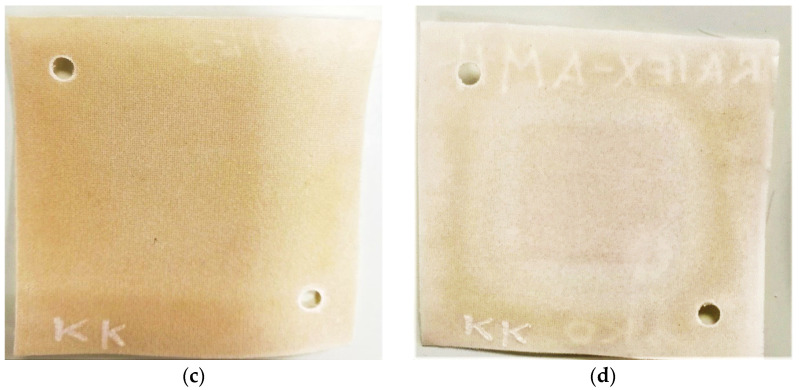
Photographs of the surfaces of MA-41P (**a**), MA-41Pmod (**b**), Ralex AMH-PES (**c**), and Ralex AMH-PESmod (**d**) anion-exchange membranes, facing the concentration chamber, after their operation in the artificial solution.

**Figure 10:**
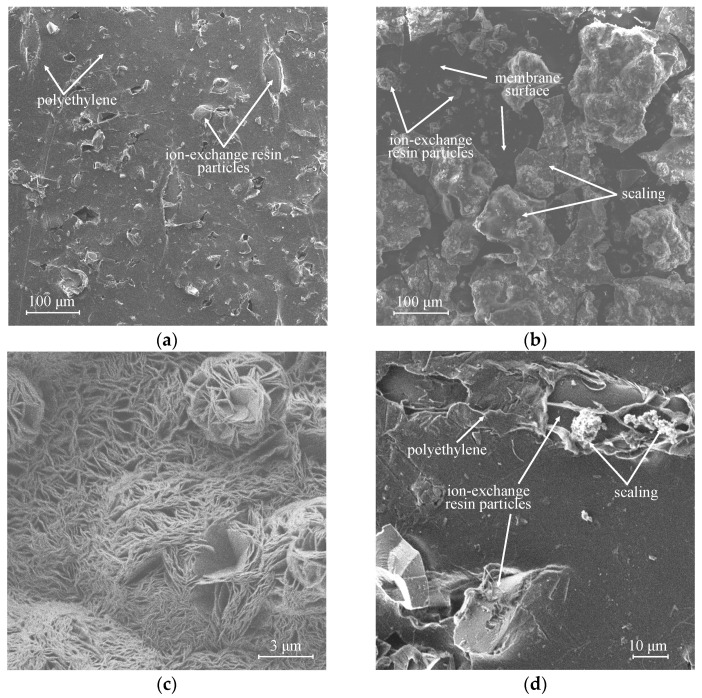

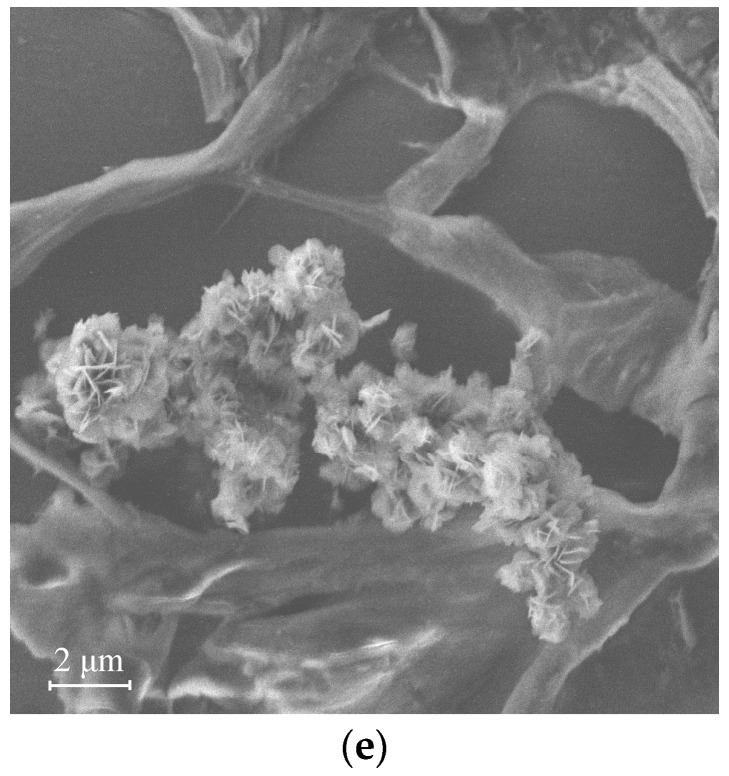
SEM images of the surface of the pristine MA-41P membrane (**a**), a scaling layer on its surface after about 5 h of electrodialysis processing of the artificial solution (**b**), and an enlarged image of the scaling morphology (**c**), as well as an image of the scaling on surface of the MA-41Pmod membrane (**d**) and enlarged image of the scaling morphology (**e**).

Even low calcium and magnesium content in the artificial solution (1% of their content in natural mine water solution) is sufficient for the scaling formation on the membrane surface during the processing of the solution by electrodialysis. Figure 9 shows optical images of the surfaces of the studied membranes (MA-41P, MA-41Pmod, Ralex AMH-PES, and Ralex AMH-PESmod) facing the concentration chamber after the electrodialysis concentration of the artificial solution was 1.25 *i*_lim_ for 5 h. It was established that the surface of the commercial MA-41P membrane after operation is covered with a dense scaling layer (Figure 9a). Previously, it was shown that magnesium hydroxide with a small amount of calcium carbonate formed on the surface [101]. Their formation is due to alkalization of the concentrate, which is associated with a high rate of water splitting when using MA-41P membrane, as shown above. The precipitate shields almost the entire surface of the heterogeneous MA-41P membrane (Figure 10a), including the areas occupied by non-conductive polyethylene (Figure 10b,c).

In contrast to the commercial MA-41P membrane, when using the MA-41Pmod (Figure 9b), as well as the Ralex AMH-PES (Figure 9c) and Ralex AMH-PESmod (Figure 9d) membranes, no visible scaling layer is formed under the same experimental conditions. However, the SEM study of the MA-41Pmod membrane surface made it possible to detect several areas with particles of magnesium hydroxide precipitate on ion-exchange particles (Figure 10d,e). In the case of both samples of Ralex, the precipitate was not detected even by SEM.

The stability of modified membrane samples is an important issue. Membrane performance should be evaluated over time to ensure the stability of the surface modification in the process. Previously, it was found that such a modified membrane is stable for at least 100 h of operation in 0.02 M NaCl in an over-limiting current mode [32]. However, for practice, the stability of the modification in multicomponent solutions is more important. We additionally tested all four membrane samples until a visible scaling appeared on their surface. More precisely, the end point of a long-term experiment was determined from the change in the period and amplitude of the potential drop oscillations, as well as from the pH value of the concentrate solution [101]. The operating conditions of the membranes were the same as for the five-hour short experiments.

It was found that while the pristine MA-41P membrane exhibits scaling resistance for only about 5 h, its modified sample MA-41Pmod is resistant to precipitation for approximately 20 h of operation in the artificial solution. In the case of the Ralex AMH-PES and Ralex AMH-PESmod membranes, the same operating time was 39 and 50 h, respectively. However, we deliberately used over-limiting current modes to speed up the degradation process. In industries, milder conditions are usually used. The longer operating time before overgrowing with sediment in the case of the last two samples is in positive agreement with the described electrochemical characteristics. Additionally, the values of the operating times are in the same row as the obtained values of the surface charge, the effective capacitance of the electric double layer, and the intensity of vortex formation: MA-41P < MA-41Pmod < Ralex AHM-PES < Ralex AHM-PESmod.

This order is explained by the high significance of the surface charge of ion-exchange membranes for both mass-transfer characteristics and resistance to precipitation [69,82,102], as well as by the stability of quaternary amino groups on the surface of ion-exchange membranes [32,103]. Indeed, as described in Section 2, MA-41P contains quaternary amino groups with a small amount of secondary and tertiary amines as functional groups, while Ralex AHM-PES contains only quaternary amino groups. The modifier interacts with all types of amino groups, because it is believed that the main mechanism of interaction is electrostatic. Accordingly, when the modifier is detached from the MA-41Pmod surface, the secondary and tertiary amino groups again begin to catalyze the water-splitting reaction [32], which leads to alkalization of the concentrate and precipitation on the surface of the anion-exchange membrane. In the case of the Ralex AHM-PESmod membrane, quaternary amino groups still remain on the membrane surface after the detachment of the modifier molecules. Changes in the electrochemical behavior of the membrane, similar to MA-41Pmod, only occur after the degradation of the amino groups initially present in the composition. The reasons for the change in the chemical nature of fixed groups are the thermohydrolysis of amino groups and the reactions proceeding according to the Hoffman and Stevens mechanisms, which are promoted by the heating of the “AEM/depleted solution” boundary and high local pH values in the membrane layer adjacent to this boundary [23,103,104].

The obtained results confirm the efficiency of membranes modified with PQ-22. The electrochemical properties are especially strongly transformed when a membrane with weakly basic amino groups is modified. An increase in the surface charge and the replacement of secondary and tertiary amino groups by quaternary ones lead to an intensification of mass transfer in over-limiting current modes and a decrease in the rate of water splitting. This, in turn, also has a positive effect on the scaling resistance.

## 4. Conclusions

The bifunctional polymer polyquaternium-22 was used to modify the surface of commercial anion-exchange membranes. It was found that the content of quaternary amino groups in PQ-22 is higher than the content of carboxyl groups. Therefore, the use of the polymer led to the nonequivalent substitution of secondary, tertiary, and even quaternary amino groups on the surface of the modified membrane. This was confirmed by voltammetry and electrochemical impedance spectroscopy, as well as by measuring the streaming potential and determining the surface charge of the modified membranes. An increase in the surface charge after membrane modification with PQ-22 promoted the intensification of electroconvection by the mechanism of electro-osmosis of the first kind. The properties of the MA-41P membrane after the modification of its surface became comparable to that of the commercial Ralex AHM-PES membrane containing only quaternary amino groups. The limiting current density of the modified MA-41P membrane increased by more than 1.5 times and the plateau length decreased by 2.5 times compared to the pristine membrane. In addition, alkalization of the diluate was observed instead of acidification after the modification of the MA-41P membrane. This means that the rate of water splitting at the adjacent cation-exchange membrane producing OH^−^ ions is higher. This is also proven by the absence of the Gerischer arc in the MA-41Pmod impedance spectra, as in the case of the Ralex AHM-PES. Thus, in the case of MA-41Pmod, electroconvection effectively developed not only by the mechanism of electro-osmosis of the first kind but also by the mechanism of electro-osmosis of the second kind. Direct visualization of the vortex structures near the surface of the membranes under study in the over-limiting current mode made it possible to establish an increase in the rate of vortex formation in the case of membranes that were in contact with the PQ-22 polymer. It was established that the modified membranes exhibit high scaling resistance. Long-term stability experiments in the artificial solution showed that the anti-scaling performance of anion-exchange membranes improves after modification. The duration of work without sedimentation with the modification of MA-41P increased from 5 to 20 h, and in the case of Ralex AHM-PES, it increased from 39 to 50 h.

## Figures and Tables

**Table 1:** Some of the characteristics of the commercial membranes under study. Results are summarized from those found in [17,64].

Membranes	Ralex AHM-PES	MA-41P
Ion-exchange groups	–N^+^(R)_3_	=NH; ≡N; –N^+^(R)_3_
Thickness in 0.02 M NaCl, μm	550 ± 3	510 ± 3
Water content, g_H_2_O_/g_dry_, %	46 ± 5	55 ± 2
Exchange capacity, mmol g^−1^ wet	1.33 ± 0.01	0.92 ± 0.05
Conductivity in 0.5 M NaCl, κ, mS/cm	6.7	10.5

**Table 2:** Zeta potential and surface charge density values of studied membranes.

Sample	Zeta Potential, ζ, mV	Surface Charge, σ, µC cm^−2^
MA-41P	14.8	0.49
MA-41Pmod	20.3	0.69
Ralex AHM-PES	21.7	0.74
Ralex AHM-PESmod	33.4	1.18

**Table 3:** Determination of the effective capacitance of the electric double layer, *C*, and the Ohmic resistance of membranes, *R*^Ώ^, from the high-frequency arc of the impedance spectra of the studied membranes at 0 A of biased DC.

Sample	$f_{\max}^{\Omega },\;\mathrm{Hz}$	−Z”, Ohm	*R*^Ώ^, Ohm	*C*, μF
MA-41P	28,189	37.6	60.3	5.6
MA-41Pmod	25,786	28.2	53.2	6.2
Ralex AHM-PES	22,182	26.0	55.2	7.2
Ralex AHM-PESmod	17,456	27.4	50.3	9.1

**Figure A1:**
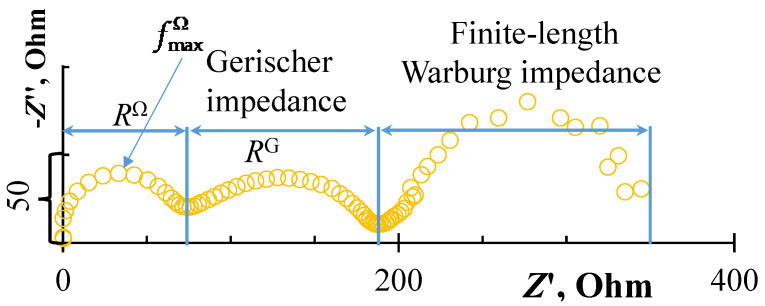
Typical impedance spectrum of a monopolar ion-exchange membrane and adjacent diffusion layers in the over-limiting current mode.

## Supplementary Materials

The following supporting information can be downloaded at: https://www.mdpi.com/article/10.3390/membranes12111065/s1, Video S1: Visualization of vortex formation at the MA-41P membrane surface in 0.02 M NaCl + 0.01 mM Rh6G at 3.0 *i*_lim_; Video S2: Visualization of vortex formation at the MA-41Pmod membrane surface in 0.02 M NaCl + 0.01 mM Rh6G at 2.7 *i*_lim_; Video S3: Visualization of vortex formation at the Ralex AMH-PES membrane surface in 0.02 M NaCl + 0.01 mM Rh6G at 2.4 *i*_lim_; Video S4: Visualization of vortex formation at the Ralex AMH-PESmod membrane surface in 0.02 M NaCl + 0.01 mM Rh6G at 2.2 *i*_lim_.

## Appendix A

### Appendix A.1. Processing of Current-Voltage Curves

The current-voltage curves (CVC) were measured in a linear galvanostatic mode. The initial current was set equal to 0 A and the final current was chosen equal to 2.5 *i*_lim_ (*i*_lim_ is the limiting current density). The current sweep rate was set to equal about 10^−6^ A/s so that the limiting current density on the membrane was reached 20–30 min after the start of the experiment. The limiting current density (*i*_lim_) was calculated using the Lévêque equation obtained within the framework of the convective-diffusion model [105]:

(A1) $$i_{\;\lim}^{}=1.47\frac{FDc_{0}}{h(T_{1}-t_{1})}{\left(\frac{h^{2}V_{0}}{LD}\right)}^{1/3}$$

where *h* is the intermembrane distance; *V*_0_ is the average linear velocity of the solution flow between the membranes; *L* is the channel length; *T*_1_ and *t*_1_ are the effective transport number of counterions (in our case, Cl^−^ ions) in the membrane and the transport number of these ions in solution, respectively; *D* is the diffusion coefficient of NaCl at infinite dilution; *F* is the Faraday constant.

When comparing current-voltage curves of different membrane systems, instead of the total potential drop, ∆*φ*, the reduced potential drop, ∆*φ*′, is used [106]:

(A2) $$\Delta \varphi^{\prime }=\Delta \varphi -iR_{0}$$

In the case of current-voltage curves, $R_{0}={\left(\partial \Delta \varphi /\partial i\right)}_{i\to 0}$ is the resistance of the membrane system when the current density, *i*, tends toward zero (found from the slope of the initial section of current-voltage curve). *R*_0_ involves the Ohmic resistance as well as the diffusion resistance due to the interfacial Donnan potential drop and diffusion potential [107].

### Appendix A.2. Processing of Electrochemical Impedance Spectra

The study of membranes by the method of electrochemical impedance spectroscopy (EIS) was also carried out in a galvanostatic mode. A selected value of direct current with an alternating current test signal of a given amplitude was set on the polarizing electrodes of the ED cell. The impedance spectrum of the membrane under study in the entire frequency range was obtained under the following measurement conditions: the set of frequencies (n = 80) was selected on a logarithmic scale in the range from 3 mHz to 500 kHz; during the impedance measurements, the frequencies were applied from low to high; the amplitude of the alternating current test signal was 250 μA. The total time taken to obtain the impedance spectrum at a given DC current value was about 1 h. The spectra were obtained at 1.25 *i*_lim_ of biased direct current.

A characteristic impedance spectrum of an IEM in a solution of strong electrolyte is shown in Figure A1. It is represented on the complex Argand plane and includes three arcs. The first arc appears at high frequencies (in the range from 10^3^ to 10^5^ Hz).

Its shape is mainly determined [96,108] by electrical capacitances and Ohmic resistances of the layers in the membrane system under study (DBL/membrane/DBL). The width of the high-frequency arc is equal to *R*^Ώ^, which is the Ohmic resistance of the membrane and the adjacent DBLs. The maximal value of the imaginary component of impedance on this arc, −Z″, and the frequency corresponding to this value, $f_{\max}^{\Omega }$, is used to estimate the effective capacitance according to equation [96]:

(A3) $$C=\frac{1}{4\pi f_{\max}^{\Omega }(-Z^{\prime \prime })}$$

This capacitance includes the capacitances of the electric double layers (EDL) on the membrane-solution boundaries, as well as the capacity caused by the asymmetry of the depleted and enriched DBLs, which occurs when a direct electric current flows. The capacity of the EDL is dominant in the given (10^3^–10^5^ Hz) frequency range [108].
